# Laser-Based Pedestrian Tracking in Outdoor Environments by Multiple Mobile Robots

**DOI:** 10.3390/s121114489

**Published:** 2012-10-29

**Authors:** Masataka Ozaki, Kei Kakimuma, Masafumi Hashimoto, Kazuhiko Takahashi

**Affiliations:** 1 Graduate School of Doshisha University, Tatara, Kyotanabe, Kyoto 6100321, Japan; E-Mails: mantuuman@gmail.com (M.O.); dtl0726@mail4.doshisha.ac.jp (K.K.); 2 Faculty of Science and Engineering, Doshisha University, Tatara, Kyotanabe, Kyoto 6100321, Japan; E-Mail: katakaha@mail.doshisha.ac.jp

**Keywords:** pedestrian tracking, multi-mobile robots, laser range scanner, Bayesian filter, decentralized multi-sensor fusion

## Abstract

This paper presents an outdoors laser-based pedestrian tracking system using a group of mobile robots located near each other. Each robot detects pedestrians from its own laser scan image using an occupancy-grid-based method, and the robot tracks the detected pedestrians via Kalman filtering and global-nearest-neighbor (GNN)-based data association. The tracking data is broadcast to multiple robots through intercommunication and is combined using the covariance intersection (CI) method. For pedestrian tracking, each robot identifies its own posture using real-time-kinematic GPS (RTK-GPS) and laser scan matching. Using our cooperative tracking method, all the robots share the tracking data with each other; hence, individual robots can always recognize pedestrians that are invisible to any other robot. The simulation and experimental results show that cooperating tracking provides the tracking performance better than conventional individual tracking does. Our tracking system functions in a decentralized manner without any central server, and therefore, this provides a degree of scalability and robustness that cannot be achieved by conventional centralized architectures.

## 1. Introduction

Tracking (*i.e.*, estimating the motion) of pedestrians is important to ensure safe navigation of mobile robots and vehicles. There has been much interest in the use of stereo vision or a laser range scanner (LRS) in mobile robotics and vehicle automation [1–5]. We previously presented a pedestrian tracking method using LRS mounted on mobile robots and automobiles [6–8].

Recently, many studies related to multi-robot coordination and cooperation have been conducted [9,10]. When these robots and vehicles are located near each other, they can share their sensing data. This implies that the robots and vehicles are considered to be a multi-sensor system. Therefore, even if pedestrians are located outside the sensing area of any individual robot or vehicle, it can detect pedestrians using the tracking data received from other robots and vehicles in the vicinity, and thus, multiple robots can improve the accuracy and reliability of pedestrian tracking.

In an intelligent transport system (ITS), if the tracking data is shared with neighboring vehicles through vehicle-to-vehicle communication, each vehicle can detect pedestrians efficiently. This facilitates the construction of an advanced driver-assistance-system. Even if pedestrians suddenly run into roads, the vehicles can detect them, and hence drivers can stop their vehicles to prevent an accident.

This paper presents a pedestrian tracking method employing multiple mobile robots and vehicles. Most studies of cooperative tracking by multiple mobile robots focus on motion planning and controlling issues [11–13]. These studies attempt to keep many moving objects visible to the mobile robots at all times while consuming as little motion energy as possible. In this paper, we address sensor-data fusion, through which pedestrian tracking is achieved by combining the tracking data from multiple mobile robots located in their vicinity.

There has been considerable research in cooperative pedestrian tracking using multiple static sensors located in the environment [14–18] and multi-sensors on robots [19,20]. Our previous work [8] presented a pedestrian tracking method using in-vehicle multi-laser range scanners; pedestrians were tracked by each LRS based on a Kalman filter. In order to enhance the tracking performance, the tracking data were blended based on covariance intersection (CI) method [21].

**Figure 1.**
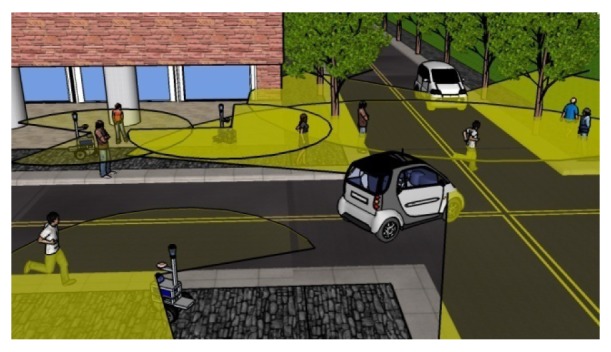
Pedestrian tracking system using multiple vehicles such as mobile robots, cars, and EPAMD.

In this paper, we extend our previous method to pedestrian tracking with multiple mobile robots in the proximity to each other. As illustrated in Figure 1, our method contributes toward building a cooperative pedestrian tracking system using vehicles such as mobile robots, cars, and electric personal assistive mobility devices (EPAMD) in future urban city environments.

Recent studies [22,23] in cooperative pedestrian tracking by multiple mobile robots require centralized data fusion with a central server; sensing data captured by each robot are sent to a central server for subsequent data fusion. The centralized data fusion reduces system robustness and scalability. Our cooperative tracking system proposed in this paper functions in a decentralized manner without any central server. This paper is organized as follows: in Section 2, we present an overview of our experimental system. In Sections 3 and 4, we present methods of pedestrian tracking and robot localization. In Section 5, we describe simulation and experiment of pedestrian tracking to validate our method, followed by our conclusions.

## 2. Experimental Mobile Robots

**Figure 2.**
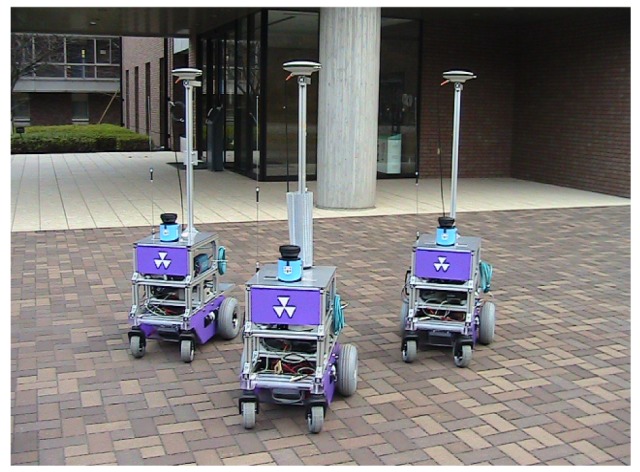
Overview of the mobile robot system.

Figure 2 shows our mobile robot system used in the experiments. We use a Okatech Mecrobot wheeled mobile robot platform. Three robots each have two independent drive-wheels. A wheel encoder is attached to each of the drive wheels to measure the wheel velocity. A fiber-optic yaw rate gyro (Tamagawa Seiki, TA7319N3) is attached to the robot's chassis to measure the turn velocity. This information is used to estimate the robot's posture based on dead reckoning. Moreover, each robot is equipped with an RTK-GPS (Novatel ProPak-V3 GPS receiver) to identify its own posture in outdoor environments. The RTK-GPS provides three types of solution: fixed, float, and single solutions. Fixed solution offers range accuracy of less than 0.2 m, and float solution achieves range accuracy of about 0.2 to 1 m. In outdoor environments causing GPS multipath problems and bad weather conditions, we get single solutions with range accuracies of several meters.

The robot is equipped with a single-layered LRS (Sick LMS100). The LRS captures laser scan images that are represented by a sequence of distance samples in a horizontal plane of 270 deg. The angular resolution of the LRS is 0.5 deg, and the number of distance samples is 541 in one scan image. The onboard computer is a Lenovo ThinkPad R500 with a 2.4 GHz Intel core 2 duo processor, and the operating system used is Microsoft Windows Vista. The sampling frequency of the sensors is 10 Hz.

Broadcast communication via a wireless LAN is used to exchange information among the robots. It takes approximately 40 ms to exchange information between robots. We employ a ring-type network structure in which the robots transmit information in the sequence: robot #1, #2, and #3.

## 3. Pedestrian Tracking

### 3.1. Overview

We define two coordinate frames: the world coordinate frame, *Σ_w_*(*O_w_* : *X_w_Y_w_*) and the *i*-th robot coordinate frame, *Σ_i_*(*O_i_* : *X_i_Y_i_*) attached at the robot body, where *i* = 1, 2, 3. Each robot independently detects pedestrians using its own laser image based on an occupancy-grid-based method. Table 1 briefly shows our occupancy grid algorithm in the pseudo-code format. Our detection method is detailed in [6,7].

**Figure 3.**
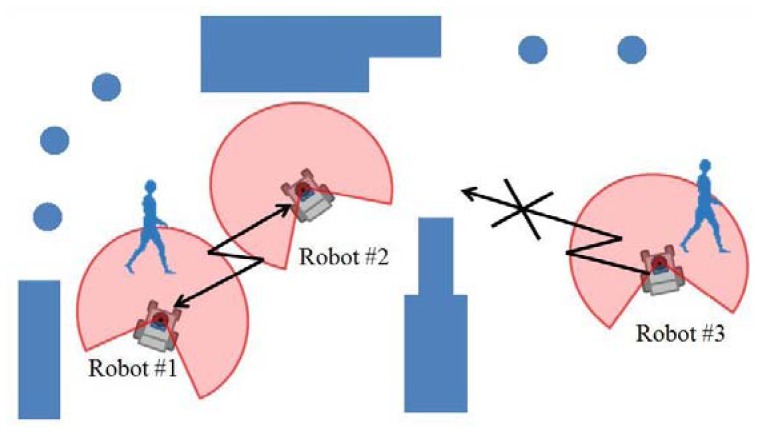
Tracking mode; robots #1 and #2 track a pedestrian in cooperative tracking mode, and robot #3 tracks a pedestrian in individual tracking mode. The red arc indicates the LRS sensing area.

The detected pedestrians are tracked using the following two tracking modes (Figure 3):

- *Individual tracking by a single robot*: Each robot individually tracks pedestrians without any tracking data from other robots. The robot can only track pedestrians inside its LRS sensing area.
- *Cooperative tracking by multiple robots*: The robots track pedestrians by sharing their own tracking data so that each robot can track pedestrians both inside and outside its LRS sensing area.

### 3.2. Individual Tracking

A pedestrian position in *Σ_w_* is denoted by (*x*,*y*). If the pedestrian is assumed to move at almost constant velocity, the rate kinematics is given by:

(1) $$\begin{array}{c} {\text{x}}_{\left(t\right)}={\text{Fx}}_{\left(t-1\right)}+\text{G}\Delta {\text{x}}_{\left(t-1\right)}\\ =\left(\begin{array}{cccc} 1 & \tau & 0 & 0\\ 0 & 1 & 0 & 0\\ 0 & 0 & 1 & \tau \\ 0 & 0 & 0 & 1 \end{array}\right){\text{x}}_{\left(t-1\right)}+\left(\begin{array}{cc} \tau^{2}/2 & 0\\ \tau & 0\\ 0 & \tau^{2}/2\\ 0 & \tau \end{array}\right)\Delta {\text{x}}_{\left(t-1\right)} \end{array}$$

where ***x*** = (*x*, *ẋ*, *y*, *ẏ*)*^T^*. *Δ**x*** = (*Δẍ*, *Δÿ*)*^T^* is an unknown acceleration (plant noise). *τ* is a sampling period of sensors; in our experimental system, *τ* is 0.1 s.

The measurement model related to the pedestrian is then:

(2) $$\begin{array}{c} {\text{z}}_{\left(t\right)}={\text{H}}_{i^{\left(t\right)}}{\text{x}}_{\left(t\right)}+{\text{H}}_{i^{\left(t\right)}}^{\prime }{\text{u}}_{i^{\left(t\right)}}+\Delta {\text{z}}_{\left(t\right)}\\ =\left(\begin{array}{cccc} \cos\psi_{i^{\left(t\right)}} & 0 & -\sin\psi_{i^{\left(t\right)}} & 0\\ \sin\psi_{i^{\left(t\right)}} & 0 & \cos\psi_{i^{\left(t\right)}} & 0 \end{array}\right){\text{x}}_{\left(t\right)}+\left(\begin{array}{cc} \cos\psi_{i^{\left(t\right)}} & -\sin\psi_{i^{\left(t\right)}}\\ \sin\psi_{i^{\left(t\right)}} & \cos\psi_{i^{\left(t\right)}} \end{array}\right){\text{u}}_{i^{\left(t\right)}}+\Delta {\text{z}}_{\left(t\right)} \end{array}$$

where ***z*** = (*z_x_*,*z_y_*)*^T^* is the measurement represented in *Σ_i_*. *Δ****z*** is the measurement noise. ***u****_i_* = (*x_i_*, *y_i_*)^*T*^ is the position of the *i*-th robot in *Σ_w_*. *ψ_i_* is the orientation of the *i*-th robot in *Σ_w_*. The posture (position and orientation) ***x****_i_* = (*x_i_*, *y_i_*, *ψ_i_*)*^T^* is determined using the localization system described in Section 4.

From Equation (1), the pedestrian's posture ***x̂*** and its associated error covariance ***P*** are predicted using a Kalman filter [24]:

(3) $$\{\begin{array}{l} {\hat{\text{x}}}_{\left(t/t-1\right)}=\text{F}{\hat{\text{x}}}_{\left(t-1\right)}\\ {\text{P}}_{\left(t/t-1\right)}=\text{F}{\text{P}}_{\left(t-1\right)}{\text{F}}^{T}+{\text{GQ}}_{\left(t-1\right)}{\text{G}}^{T} \end{array}$$

where ***Q*** is the covariance of the plant noise *Δ**x***.

**Figure 4.**
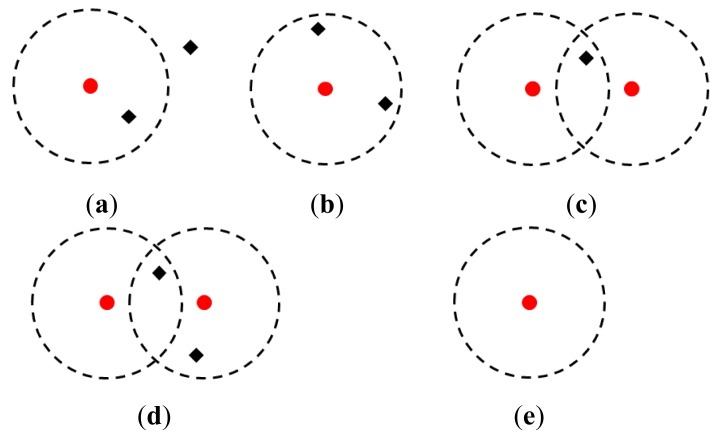
Tracking condition; the red circle and black diamond indicate the tracked pedestrian and the measurement, respectively. The dashed circle indicates the validation region. (**a**) Case 1. (**b**) Case 2. (**c**) Case 3. (**d**) Case 4. (**e**) Case 5.

To track multiple pedestrians, as shown in Figure 4(a), a validation region with a constant radius is set around the predicted position (***x****^*, *ŷ*) of each tracked pedestrian. The measurements inside the validation region are considered to be obtained from the tracked pedestrian, and it is applied to the track updated with the Kalman filter. On the other hand, the measurements outside the validation region are considered to be false alarms, and are therefore, discarded. From Equations (2) and (3), the posture of the tracked pedestrian and its associated error covariance are updated by:

(4) $$\{\begin{array}{c} {\hat{\text{x}}}_{\left(t\right)}={\hat{\text{x}}}_{\left(t/t-1\right)}+{\text{K}}_{\left(t\right)}({\text{z}}_{\left(t\right)}-{\text{H}}_{i^{\left(t\right)}}{\hat{\text{x}}}_{\left(t/t-1\right)}+{\text{H}}_{i^{\left(t\right)}}^{\prime }{\text{u}}_{i^{\left(t\right)}})\\ {\text{P}}_{\left(t\right)}={\text{P}}_{\left(t/t-1\right)}+{\text{K}}_{\left(t\right)}{\text{H}}_{i^{\left(t\right)}}{\text{P}}_{\left(t/t-1\right)} \end{array}$$

where ***K***_(_*_t_*_)_ = ***P***_(_*_t_*_/_*_t_*_−1)_***H***_*i*_(*t*)__*^T^****S***_(_*_t_*_/_*_t_*_−1)_^−1^, and ***S***_(_*_t_*_/_*_t_*_−1)_ = ***H***_*i*^(*t*)^_***P***_(_*_t_*_/_*_t_*_−1)_***H***_*i*^(*t*)^_*^T^* + ***R***_(_*_t_*_)_. ***R*** is the covariance of the measurement noise *Δ**z***.

In our simulation and experiment described in Section 5, the radius of the validation region is set at 1.0 m. The covariances of the plant and measurement noises in Equations (3) and (4) are set at ***Q*** = diag (1.0 m^2^/s^4^, 1.0 m^2^/s^4^) and ***R*** = diag (0.01 m^2^, 0.01 m^2^), respectively.

In crowded environments, as shown in Figures 4(b–d), multiple measurements exist inside a validation region; multiple tracked pedestrians also compete for measurements. To achieve a reliable data association (matching of tracked pedestrians and measurements), we apply a global-nearest-neighbor (GNN) algorithm [25].

We consider that, in a validation region, *J* pedestrians exist and *K* measurements are received, where *J* does not necessarily equal *K*. We then define the distance measure *λ_jk_* from the *j*-th tracked pedestrian to the *k*-th measurement, where *j* = 1,2, …, *J* and *k* = 1,2, …, *K* as:

(5) λjk=(zk(t)−u^j(t/t−1))T(Sj(t/t−1))(zk(t)−u^j(t/t−1))−1

where ***S***_*j*^(*t*/*t*−1)^_ = ***H***_*j*^(*t*)^_***P***_*j*^(*t*/*t*−1)^_***H***_*j*^(*t*)^_*^T^* + ***R***(*t*), and ***u***_*j*^(*t*/*t*−1)^_ is the predicted position of the *j*-th tracked pedestrian.

We then define the following cost matrix ***Λ***:

(6) $$\text{Λ}=\left(\begin{array}{cccc} \lambda_{11} & \lambda_{12} & \cdots & \lambda_{1K}\\ \lambda_{21} & \lambda_{22} & \cdots & \lambda_{2K}\\ \vdots & \vdots & \ddots & \vdots \\ \lambda_{J1} & \lambda_{J2} & \cdots & \lambda_{JK} \end{array}\right)$$

We assume that the *a*(*j*)-th measurement is assigned to the *j*-th pedestrian. The data association is achieved by finding the *a*(*j*) based on the Munkres algorithm [26] so that $\sum_{j=1}^{J}\lambda_{ja(j)}$ can be minimized. It is noted that if the *k*-th measurement does not exist inside the validation of the *j*-th tracked person, we set the distance measure at *λ_jk_* = ∞.

Pedestrians always appear in and disappear from the LRS sensing area. They also face interaction and occlusion issues. In order to handle such conditions, we implement a tracking management system based on the following rules:

- *Track initiation*: As shown in Figures 4(a–c), the measurements that are not matched with any tracked pedestrians are considered to come from new pedestrians or false alarms, which disappear soon. Therefore, we tentatively initiate tracking of the measurements with Kalman filter. If the measurements are always visible in more than *N*_1_ s, they are considered to come from new pedestrians, and the tracking is continued. If the measurements disappear within *N*_1_ s, they are considered to be the false alarms, and the tentative tracking is terminated.
- *Track termination*: When the tracking pedestrians exit the sensing area of the LRS or they meet occlusion, no measurements exist within their validation regions. If no measurements arise from the temporal occlusion, the measurements appear again. We thus predict the positions of the tracking pedestrians with the Kalman filter. If the measurements appear again within *N*_2_ s, we proceed with the tracking. Otherwise (see Figure 4(e)), we terminate the tracking. In our simulation and experiment described in Section 5, we set *N*_1_ = 1.5 and *N*_2_ = 3.0 by trial and error.

For simplicity, in this paper, pedestrians are assumed to move at an almost constant velocity, and they are tracked using the usual Kalman filter. If the pedestrians move randomly, such as walking, running, going or stopping suddenly, and turning suddenly, using multi-model-based tracking can improve the tracking performance [14,15].

### 3.3. Cooperative Tracking

When the robots are located near to each other, the tracking mode is switched to cooperative tracking. They communicate with each other and exchange their own tracking data, which consist of estimated positions and velocities of tracked pedestrians and their associated error covariances. Because tracking data are shared, each individual robot constantly tracks pedestrians both inside and outside its own LRS sensing area.

**Figure 5.**
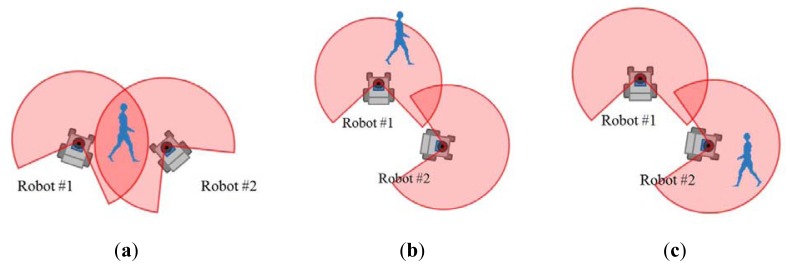
Conditions in cooperative tracking. (**a**) Case 1. (**b**) Case 2. (**c**) Case 3.

To elucidate cooperative tracking in detail, we consider two robots #1 and #2, as shown in Figure 5. The tracking data for the *m*-th pedestrian tracked by robot #1 is denoted by ${\text{I}}_{m}^{\left(1\right)}=\{{\hat{\text{x}}}_{m}^{\left(1\right)},{\text{P}}_{m}^{\left(1\right)}\}$, where *m* = 1,2, …. ${\hat{\text{x}}}_{m}^{\left(1\right)}$ denotes the estimate (position estimate ${\hat{\text{q}}}_{m}^{\left(1\right)}$ and velocity estimate ${\hat{\dot{\text{q}}}}_{m}^{\left(1\right)}$); ${\text{P}}_{m}^{\left(1\right)}$ is its associated error covariance. Similarly, the tracking data for the *n*-th pedestrian tracked by robot #2 is denoted by ${\text{I}}_{n}^{\left(2\right)}=\{{\hat{\text{x}}}_{n}^{\left(2\right)},{\text{P}}_{n}^{\left(2\right)}\}$, where *n* = 1,2, …. We consider that robot #1 combines the tracking data sent from robot #2 with its own tracking data. Combining the tracking data of robot #1 with that of robot #2 can be achieved similarly.

First, we set a validation region with a constant radius around the position estimate ${\hat{\text{q}}}_{m}^{\left(1\right)}$ of the *m*-th pedestrian tracked by robot #1. We consider the position estimate ${\hat{\text{q}}}_{n}^{\left(2\right)}$ of the *n*-th pedestrian tracked by robot #2 as the measurement, and then, we can determine data association (one-to-one matching of pedestrians tracked by robots #1 and #2) using the GNN algorithm. The GNN-based data association in cooperative tracking is similar as one in individual tracking mentioned in Section 3.1. In our simulation and experiment described in Section 5, the radius of the validation region is set at 1.2 m.

As shown in Figure 5(a), when a pedestrian is detected inside the sensing areas of both robots #1 and #2, the two estimates, ***q̂***^(1)^ and ***q̂***^(2)^, of the pedestrian can be matched. For the matched pedestrian, robot #1 updates its own tracking data by the CI method [21]:

(7) $$\left\{ \begin{array}{l} {\hat{\text{x}}}_{\left(t\right)}^{(1)+}={\text{P}}_{\left(t\right)}^{(1)+}\left\{\omega {\text{P}}_{\left(t\right)}^{(1)-1}{\hat{\text{x}}}_{\left(t\right)}^{\left(1\right)}+(1-\omega ){\text{P}}_{\left(t\right)}^{(2)-1}{\hat{\text{x}}}_{\left(t\right)}^{\left(2\right)}\right\}\\ {\text{P}}_{\left(t\right)}^{(1)+}={\left\{\omega {\text{P}}_{\left(t\right)}^{(1)-1}+(1-\omega ){\text{P}}_{\left(t\right)}^{(2)-1}\right\}}^{-1} \end{array}\right.$$

where ***I***^(1)^ = {***x̂***^(1)^, ***P***^(1)^} and ***I***^(2)^ = {***x̂***^(2)^, ***P***^(2)^} denote the tracking data of the matched pedestrian. ***x̂***^(1)+^ and ***P***^(1)+^ denote the updated tracking data and its associated error covariance, respectively. The weight *ω* is selected using the Golden selection search (GSS) method so that the determinant of ***P***^(1)+^ can be minimized under the constraint 0 ≤ *ω* ≤ 1. In simulation and experiment described in Section 5, the convergence threshold of the weight *ω* is set at 1.0*10^−4^. In this case, we can determine the appropriate weight *ω* by iterative calculation of less than twenty times.

**Figure 6.**
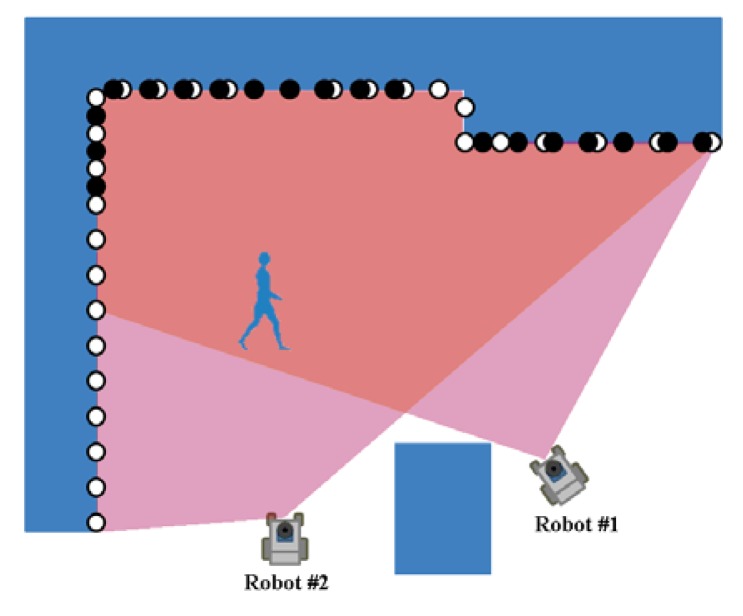
Robot localization by relative-scan matching; the solid and open circles indicate images scanned by robots #1 and #2, respectively.

As shown in Figure 5(b,c), for non-matched pedestrian, robot #1 updates its own tracking data as follows:

- When a pedestrian appears inside the sensing area of robot #1 but outside that of robot #2, as shown in Figure 5(b), robot #1 has the tracking data ***I***^(1)^, but robot #2 does not have ***I***^(2)^. Then, robot #1 sets ***I***^(1)+^ = ***I***^(1)^.
- When a pedestrian appears inside the sensing area of robot #2 but outside that of robot #1 as shown in Figure 6(c), robot #2 has the tracking data ***I***^(2)^, but robot #1 does not have ***I***^(1)^. Then, robot #1 sets ***I***^(1)+^ = ***I***^(2)^.

Cooperative tracking with three or more robots can be achieved in a similar manner. Decentralized data fusion provides better system scalability and reliability than centralized data fusion [21]. Therefore, we combine the tracking data in a decentralized manner. Statistically, the tracking data are highly correlated. The conventional Kalman filter-based fusion hampers the development of a decentralized system because it needs to calculate the degree of their correlation. The CI method allows accurate fusion of the tracking data in a decentralized manner without the knowledge of the degree of their correlation. Therefore, we apply the CI algorithm.

Data association is important in pedestrian tracking. In this paper, we apply GNN-based data association to match the current measurement scan to the existing tracks. An alternative effective data association algorithm is multiple hypothesis tracking (MHT) [27,28]. In MHT, the feasible measurement-to-track association hypotheses are enumerated and evaluated up to a certain time depth. The MHT-based data association may outperform the GNN data association in crowded environments; however, in our experience, MHT data association makes real-time tracking difficult in crowded environments because it requires the evaluation of an exponentially increasing number of feasible data association hypotheses. The MHT data association also requires centralized data fusion with a central server [22]. Therefore, we apply GNN data association.

## 4. Estimation of Robot Posture

To achieve cooperative tracking, each robot must always identify its own posture (position and orientation) with a high degree of accuracy in a world coordinate frame *Σ_w_* and map the tracking data onto *Σ_w_*, for which we apply RTK-GPS. The robot also determines its own posture by a scan matching based localization to improve the accuracy of its posture. If the robot cannot retrieve RTK-GPS information, only the scan matching based localization is applied to determine its own posture.

### 4.1. RTK-GPS Based Localization

The robot estimates its own velocity (linear/turning velocity) based on dead reckoning using the wheel encoders and gyro. The robot is assumed to move at nearly constant velocity. Motion and measurement models of the *i*-th robot are then given by Equations (8) and (9), respectively:

(8) $${\text{V}}_{i^{\left(t\right)}}={\text{V}}_{i^{\left(t-1\right)}}+\Delta {\text{V}}_{i^{\left(t-1\right)}}$$

(9) $${\text{z}}_{i^{\left(t\right)}}=\left(\begin{array}{cc} 1 & -b/2\\ 1 & b/2\\ 0 & 1 \end{array}\right){\text{V}}_{i^{\left(t\right)}}+\Delta {\text{z}}_{i^{\left(t\right)}}$$

where ***V****_i_* = (*v_i_*, *ψ̇_i_*)*^T^*; *v_i_* is the linear velocity and *ψ̇_i_* is the turning velocity. ***z****_i_* = (*z_l_*, *z_r_*, *z_ψ_*)*^T^*; ***z****_l_* and *z_r_* are the velocities of the left and right wheels, respectively, measured by the wheel encoders , and *z_ψ_* is the gyro output. *Δ**V**_i_* and *Δ**z**_i_* are unknown acceleration (disturbance) and the sensor noise, respectively. *b* is the tread length of the robot.

From Equations (8) and (9), the robot velocity ***V****_i_* is estimated using Kalman filter. Based on the velocity estimate ***V̂****_i_*, we can determine the posture of the *i*-th robot ***x****_i_* = (*x_i_*, *y_i_*, *ψ_i_*)*^T^* and its associated covariance by Equations (10) and (11), respectively:

(10) $${\hat{\text{x}}}_{i^{\left(t/t-1\right)}}=\left(\begin{array}{c} {\hat{x}}_{i^{\left(t-1\right)}}+{\hat{V}}_{i^{\left(t-1\right)}}\tau \cos({\hat{\psi }}_{i^{\left(t-1\right)}}+\frac{{\hat{\dot{\psi }}}_{i^{\left(t-1\right)}}}{2}\tau )\\ {\hat{y}}_{i^{\left(t-1\right)}}+{\hat{V}}_{i^{\left(t-1\right)}}\tau \sin({\hat{\psi }}_{i^{\left(t-1\right)}}+\frac{{\hat{\dot{\psi }}}_{i^{\left(t-1\right)}}}{2}\tau )\\ {\hat{\psi }}_{i^{\left(t-1\right)}}+{\hat{\dot{\psi }}}_{i^{\left(t-1\right)}}\tau \end{array}\right)$$

(11) $${\text{P}}_{i^{\left(t/t-1\right)}}=\nabla {\text{f}}_{\left(t-1\right)}{\text{P}}_{i^{\left(t-1\right)}}\nabla {{\text{f}}_{\left(t-1\right)}}^{T}+\nabla {{\text{f}}^{\prime }}_{\left(t-1\right)}{\text{Q}}_{i^{\left(t-1\right)}}\nabla {{{\text{f}}^{\prime }}_{\left(t-1\right)}}^{T}$$

where ***x̂***_*i*^(*t*/*t*−1)^_ and ***P***_*i*^(*t*/*t*−1)^_ are the posture estimate and its error covariance, respectively. ***Q****_i_* is the error covariance of ***V̂****_i_*. ∇***f*** and ∇***f′*** are the Jacobian matrices of Equation (10) at ***x̂***_*i*^(*t*/*t*−1)^_ and ***V̂****_i_*, respectively.*τ* is a sampling period of sensors.

The measurement model related to the RTK-GPS is given by:

(12) $$\begin{array}{c} {\text{z}}_{{\text{GPS}}^{\left(t\right)}}=\text{H}{\text{x}}_{i^{\left(t\right)}}+\Delta {\text{z}}_{{\text{GPS}}^{\left(t\right)}}\\ =\left(\begin{array}{ccc} 1 & 0 & 0\\ 0 & 1 & 0 \end{array}\right){\text{x}}_{i^{\left(t\right)}}+\Delta {\text{z}}_{{\text{GPS}}^{\left(t\right)}} \end{array}$$

where ***z****_GPS_* is the measurement; position of the *i*-th robot in *Σ_w_*, and *Δ**z**_GPS_* is the measurement noise.

If the robot obtains posture information from the RTK-GPS, the robot can update its own posture and its associated covariance using Kalman filter as follows:

(13) $$\{\begin{array}{l} {\hat{\text{x}}}_{i^{\left(t\right)}}={\hat{\text{x}}}_{i^{\left(t/t-1\right)}}+{\text{K}}_{\left(t\right)}[{\text{z}}_{{\text{GPS}}^{\left(t\right)}}-\text{H}{\hat{\text{x}}}_{i^{\left(t/t-1\right)}}]\\ {\text{P}}_{i^{\left(t\right)}}={\text{P}}_{i^{\left(t/t-1\right)}}-{\text{K}}_{\left(t\right)}\text{H}{\text{P}}_{i^{\left(t/t-1\right)}} \end{array}$$

where ***K***_(_*_t_*_)_ = ***P***_*i*^(*t*/*t*−1)^_***H***_(_*_t_*_)_*^T^* (***HP***_*i*^(*t*/*t*−1)^_***H****^T^* + ***R***_*GPS*^(*t*)^)_^−1^, and ***R****_GPS_* is the covariance of the measurement noise *Δ**z**_GPS_*.

### 4.2. Scan Matching Based Localization

When multiple robots are located near each other, they have an overlapping sensing area. They improve their own posture accuracy by exchanging their laser-scan images and matching them in their overlapping sensing area.

To elucidate scan matching based localization in detail, we consider two robots #1 and #2, as shown in Figure 6, where the two robots are located near each other and their sensing areas partially overlap. We define the posture of robot #2 relative to robot #1 by ^2^***z***_1_ = (^2^*x*_1_,^2^*y*_1_,^2^*ψ*_1_)*^T^* in *Σ_w_*. Robot #1 broadcasts its own posture and a laser scan image obtained by its own LRS to robot #2. Robot #2 determines the relative posture, ^2^***z***_1_, by matching its own laser scan image with that sent from robot #1 (Appendix). Hereafter, we call the laser scan matching for estimating relative posture as *relative-scan matching*.

Measurement model related to the relative-scan matching is given by:

(14) z21(t)=g(x^1(t),x2(t))+Δz21(t)=(cosψ2(t)−sinψ2(t)0sinψ2(t)cosψ2(t)0001)(x^1(t)−x2(t)y^1(t)−y2(t)ψ^1(t)−ψ2(t))+Δz21(t)

where ***x̂***_1_ is the posture of robot #1 estimated by the RTK-GPS-based localization. *Δ*^2^***z***_1_ is the error of the relative posture. From Equations (13) and (14), robot #2 can determine its own posture ***x̂***_2_ and its associated error covariance ***P***_2_ using Kalman filter:

(15) {x^2(t)=x^2(t/t−1)+K(t)[z21(t)−g(x^1(t),x^2(t/t−1))]P2(t)=P2(t/t−1)−K(t)∇g(t)P2(t/t−1)

where ***K***_(*t*)_ = ***P***_2^(*t*/*t*−1)^_∇***g***_(*t*)_*^T^* (∇***g***_(*t*)_***P***_2^(*t*/*t*−1)^_**∇*g***_(*t*)_*^T^*+^2^***R***_1^(*t*)^_)^−1^. ∇***g*** is the Jacobian matrix of ***g*** in Equation (14) at ***x̂***_2^(*t*/*t*−1)^_, and ^2^***R***_1_ is covariance of *Δ*^2^***z***_1_.

## 5. Simulation and Experimental Results

### 5.1. Simulation Results

**Figure 7.**
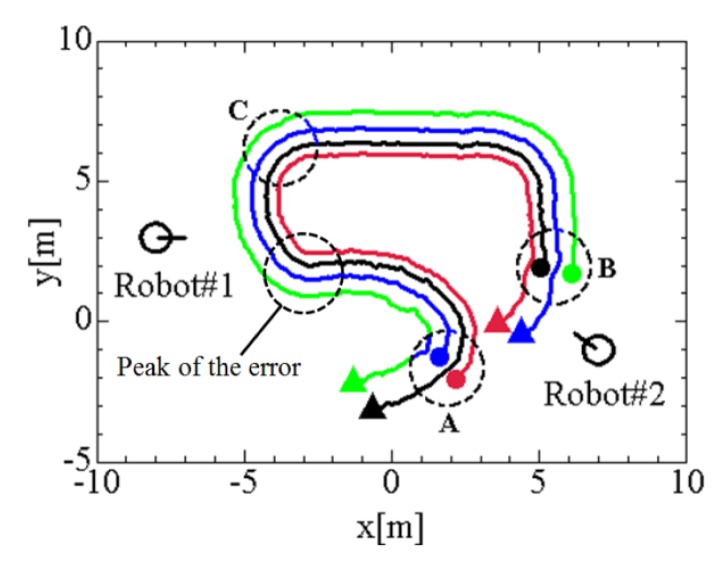
Simulation condition. Red, blue, green and black lines indicate moving path of pedestrians #1, #2, #3 and #4, respectively.

In the experiment described in Section 5.2, it is very difficult to recognize the true positions of the tracked pedestrians. Therefore, we evaluate the performance of the proposed method by simulating four-pedestrian tracking by two robots. As shown in Figure 7, two robots stops at the coordinates (*x*,*y*)=(−8.0,3.0)m and (7.0,−1.0)m, and pedestrians move at the velocity of 0.1–1.7 m/s; pedestrians #1 and #2 move side-by-side at the distance of 0.8 m from start point A, and pedestrians #3 and #4 move side-by-side at the distance of 0.8 m from start point B. Four pedestrians meet each other at point C. In the simulation, the pedestrians are assumed to be always detected correctly, and measurement noise of LRS is assumed to be uniform distribution between −0.05 m and 0.05 m. Simulation tool/software is self-produced using C++ language.

**Figure 8.**
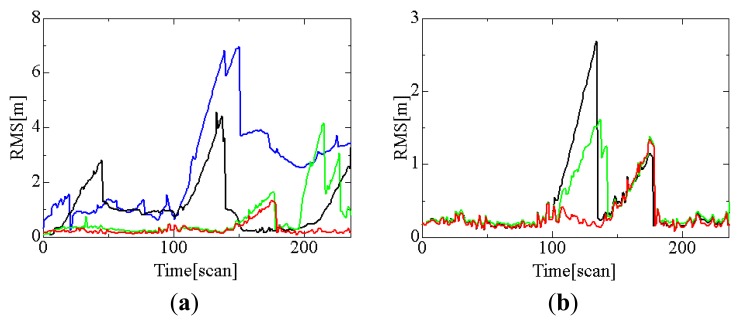
Tracking error. (**a**) Effect of data association method and tracking mode. (**b**) Effect of data fusion method. In (a), red, green, blue and black lines indicate the results by cooperative tracking using GNN, individual tracking using GNN, cooperative tracking using NN, and individual tracking using NN, respectively. In (b), red, green and black lines indicate the results by CI, Kalman filter and averaging method, respectively.

Figure 8(a) shows the effect of tracking mode and data association methods to the tracking error; GNN and conventional nearest neighbor (NN) [24] methods are applied for the data association. Tracking error is evaluated by the following root mean squared error (RMS):

(16) $${\text{RMS}}_{\left(t\right)}=\sqrt{\frac{1}{4}\overset{4}{\underset{i=1}{\text{∑}}}{\left({\hat{\text{u}}}_{i^{\left(t\right)}}-{\text{u}}_{i^{\left(t\right)}}\right)}^{T}({\hat{\text{u}}}_{i^{\left(t\right)}}-{\text{u}}_{i^{\left(t\right)}})}$$

where ***û***_*i*^(*t*)^_=(*x̂*_*i*^(*t*)^_, *ŷ*_*i*^(*t*)^_)*^T^* and ***u***_*i*^(*t*)^_=(*x*_*i*^(*t*)^_, *y*_*i*^(*t*)^_)*^T^* denote the position estimate and the true position, respectively, of the *i*-th pedestrian, where *i* = 1, 2, 3, 4, at the *t*-th laser scan.

First of all, we compare the result of cooperative tracking by GNN data association with that of individual tracking by GNN data association. Both tracking modes have the similar tracking error before 200 scan (20 s); however, individual tracking mode causes large tracking error after 200 scan. Because pedestrian #2 is shadowed by pedestrian #1 around 183 scan, robot #1 loses pedestrian #2 and large tracking error occurs in individual tracking mode after 200scan. However, pedestrian #2 is visible by robot #2 even around 183 scan, and thus cooperative tracking can maintain the accurate tracking after 200scan.

Both tracking modes temporarily cause large tracking error around 160 scan (around (*x*,*y*) = (−3.0,0.7)m in Figure 7). This reason is why pedestrian #3 is temporarily shadowed by pedestrian #4 and track lost then occurs.

NN-based data association very often causes incorrect matching of tracked pedestrians and LRS measurements, and this results in the track being lost. On the other side, GNN data association reduces the track lost. Therefore, the tracking error by GNN data association becomes smaller than that by NN data association. As the result, it is clear from Figure 8(a) that cooperative tracking by GNN data association provides better tracking performance than other methods.

Next, we simulate the effect of the data fusion methods for the cooperative tracking to the tracking error. For comparison purpose, we consider three data fusion methods: CI method, Kalman filter, and averaging method. In the Kalman filter, data fusion is achieved by considering the tracking data sent from other robots to be measurements. Based on the averaging method, each robot tracks pedestrians by simply averaging its own tracking data with the tracking data sent from other robots; the averaging method equals CI method by setting the weight *ω* = 0.5. In this simulation, GNN data association is always applied for the data association.

Figure 8(b) shows the results. The Kalman filter and averaging method cause large tracking errors around 120 scans (around point C in Figure 7). The data fusion method is closely related to the data association method; the performance in data fusion affects that in data association, and vice versa. Compared to Kalman filter and average methods, CI method maintains the accurate tracking performance. From these simulations, we confirmed that cooperative tracking based on CI and GNN methods provides tracking performance better.

### 5.2. Experimental Results

**Figure 9.**
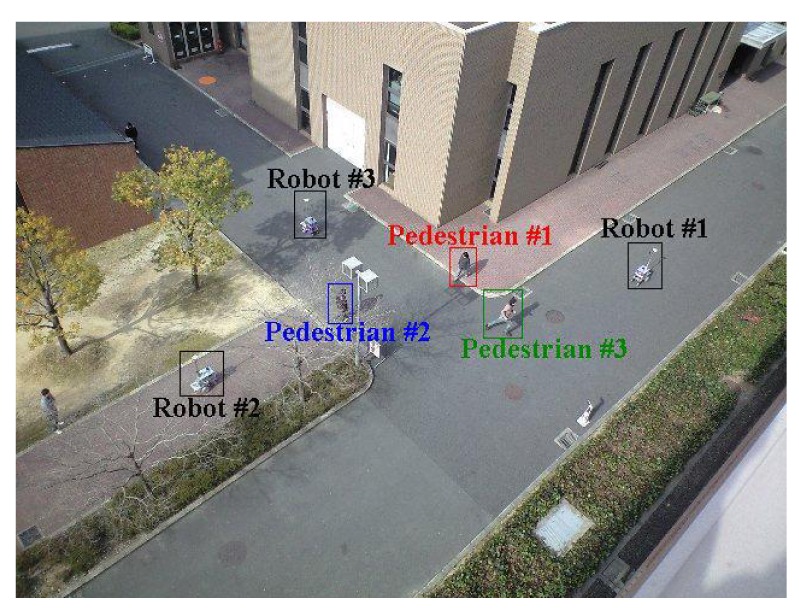
View of experimental environments.

**Figure 10.**
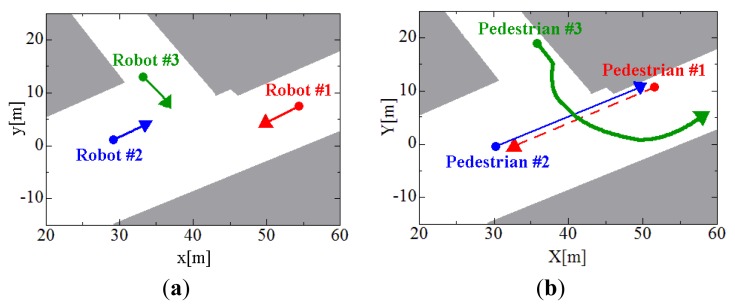
Movement path of robots and pedestrians. (**a**) Robot path. (**b**) Pedestrian path.

To evaluate the tracking method, we conducted an experiment in an outdoor environment shown in Figure 9. Three robots and three pedestrians move around in the environment as shown in Figure 10. The moving speed of the robots is less than 0.3 m/s. The walking speeds of pedestrians #1, #2, and #3 are less than 1.5 m/s, 1.5 m/s, and 3.7 m/s, respectively: At first, pedestrian #3 walks at the same speed of pedestrians #1 and #2, and he runs at a speed of 3.7 m/s on the way. The experimental time is 188 scans (18.8 s).

**Figure 11.**
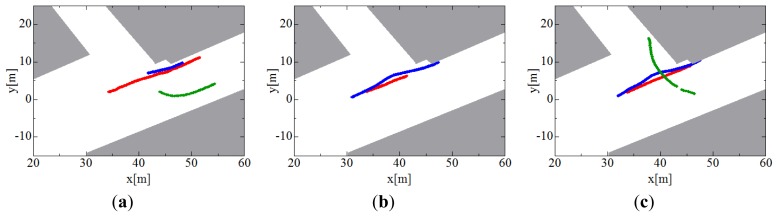
Pedestrian tracks estimated by individual tracking. (**a**) Robot #1. (**b**) Robot #2. (**c**) Robot #3. Red, blue and green lines indicate paths of pedestrians #1, #2 and #3, respectively.

Figure 11 shows the results of pedestrian tracking only by individual tracking; figures (a), (b) and (c) show the tracks of three pedestrians estimated by robot #1, #2, and #3, respectively. Each robot partially tracks pedestrians because the pedestrians exist inside and outside the sensing area of the LRS.

**Figure 12.**
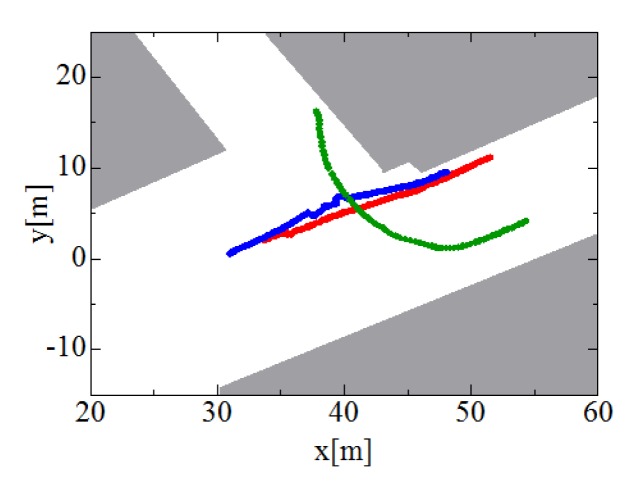
Pedestrian tracks estimated by individual and cooperative tracking. Red, blue and green lines indicate paths of pedestrians #1, #2 and #3, respectively.

Figure 12 shows the tracks of three pedestrians estimated by individual and cooperative tracking; because the three robots share the tracking data with each other, all three robots can track the three pedestrians for an extended period.

**Figure 13.**
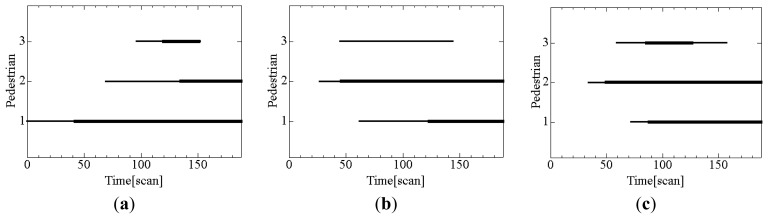
Duration of individual tracking. (**a**) Robot #1. (**b**) Robot #2. (**c**) Robot #3. The thin line indicates the time during which the pedestrian exits the sensing area of the robot. The bold line indicates the time during which the robot tracks pedestrians using the individual tracking.

**Figure 14.**
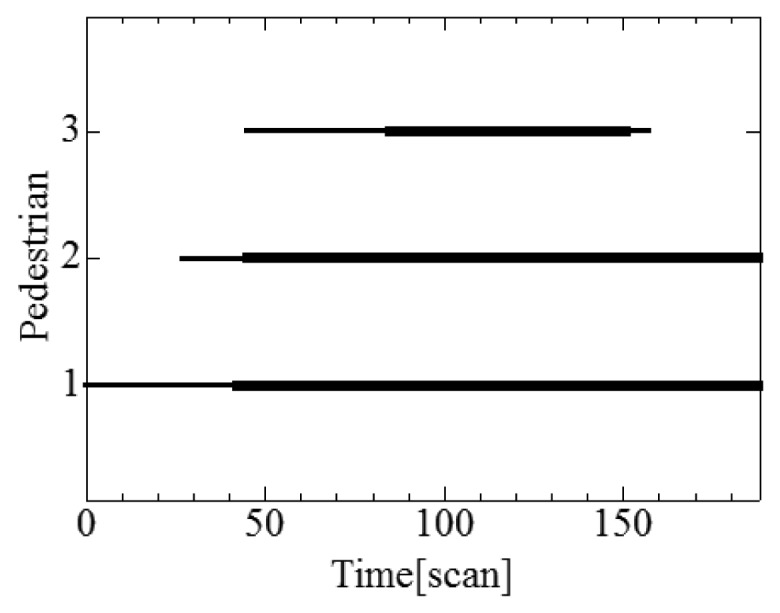
Duration of individual and cooperative tracking. The thin line indicates the time during which the pedestrian exits the sensing area of each robot. The bold line indicates the time during which the robot tracks pedestrians using the individual and cooperative tracking.

Figure 13 shows the duration of pedestrian tracking; figures (a), (b) and (c) show the times during which robots #1, #2 and #3, respectively, track pedestrians using individual tracking. Figure 14 shows the duration of pedestrian tracking by the individual and cooperative tracking. From these results, cooperative tracking provides a better tracking performance than individual tracking; for example, cooperative tracking detects pedestrian #3 who runs into the road 34 scan (3.4 s) faster than individual tracking. The faster the pedestrians can be detected, the safer becomes robot's navigation.

## 6. Conclusions

This paper presents a laser-based pedestrian tracking method using multiple mobile robots. Pedestrians were tracked by each robot using Kalman filter and GNN based data association. The tracking data obtained by each robot was broadcast to others robots and was combined by the CI method. Our method shares the pedestrian tracking data with all robots, and thus, collectively they can always recognize pedestrians that may be invisible to individual robots. The method was validated by simulation and experiment. Our tracking system worked effectively in a decentralized manner without any central server.

In the experiment, three pedestrians were tracked in a sparse environment. We will next conduct pedestrian tracking experiments in crowded environments. To achieve cooperative tracking, the robots must always identify their own postures with a high degree of accuracy in a common coordinate frame, for which, in this paper, we applied two localization methods: RTK-GPS-based and relative-scan matching-based. However, in outdoor environments such as areas surrounded by high buildings and roadside trees, it is difficult for robots to obtain posture information accurately by GPS due to GPS multipath and diffraction problems and so on. To cope with this problem, we will embed a simultaneously localization and mapping (SLAM) method into our tracking system; SLAM-GPS fusion based localization will always maintain a high degree of positioning accuracy, and therefore, it will enhance the robustness of our cooperative pedestrian tracking system in GPS-denied environments such as urban cities.

## Figures and Tables

**Figure 15.**
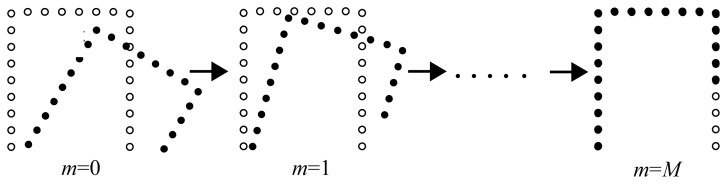
Relative-scan matching. Solid and open circles indicate scan images taken by robots #1 and #2, respectively.

**Table 1.** Occupancy grid algorithm.

Let *C* [*X*_max_, *Y*_max_] be a two dimensional array of cells counting the number of observations, where *X*_max_ and *Y*_max_ are the maximum *X* and *Y* coordinates. Intialize all cells in *C* to zero. Make an observation with laser range scanner. Determine which cells in *C* are occupied in the current laser scan image, and increment the occupied cells *C* [*X*, *Y*]. If *C* [*X*, *Y*] == 0, then we have no information on the cell—free space. If *C* [*X*, *Y*] ≧ 7, then the cell is “static cell”—static object. If 0 < *C* [*X*, *Y*] < 7, then the cell is “moving cell”—moving object; pedestrian. Repeat from step 3.

## Appendix

### Relative-Scan Matching

We determine the posture of robot #2 ^2^***z***_1_ = (^2^*x*_1_,^2^*y*_1_,^2^*ψ*_1_)*^T^* relative to that of robot #1 based on laser-scan matching method. The matching is based on point-to-point scan matching by the iterative closest point (ICP) algorithm [29].

From the laser scan images taken by robots #1 and #2, we compute the relative posture ^2^***z***_1_ using the weighted least-squares method; the cost function is given as:

(A1) $$J={\overset{541}{\underset{i=1}{\text{∑}}}w_{i}\{{\text{q}}_{j}-(\text{R}{\text{p}}_{i}+\text{T})\}}^{2}$$

where $\text{R}=\left(\begin{array}{cc} \cos^{2}\psi_{1} & -\sin^{2}\psi_{1}\\ \sin^{2}\psi_{1} & \cos^{2}\psi_{1} \end{array}\right)$ and T=(x21y21).

Here, ***p****_i_* = (*p_xi_*, *p_yi_*)*^T^* , where *i* = 1, 2, …, 541, denotes the distance sample (scan image) from robot #1; ***q****_j_* = (*q_xj_*, *q_yj_*)*^T^*, where *j* = 1, 2, … 541, denotes the distance sample from robot #2, as shown in Figure A1.

Each sample ***p****_i_* corresponds to the minimum distance sample ***q****_j_* of all samples in the scan by robot #2; *w_i_* denotes the weight. ***R*** and ***T*** denote the rotational matrix and translational vector, respectively.

To reduce the effects of correspondence errors in the distance samples in the two laser images, we define weight *w_i_* according to the errors between correspondence points *w_i_* = (1+*d_ij_* /*C*)^−1^, where *d_ij_* denotes the distance error between the two laser images and *C* is a constant. In our experiment described in Section 5, *C* is set at 0.1.

From Equation (A1), the iterative least-squares method is used to update the relative posture z21(m−1)as follows:

(A2) z21(m)=z21(m−1)+(HTWH)−1HTW(q−p)

where $\text{p}={\left({\text{p}}_{1}^{T},{\text{p}}_{2}^{T},\ldots ,{\text{p}}_{541}^{T}\right)}^{T}$, $\text{q}={\left({\text{q}}_{1}^{T},{\text{q}}_{2}^{T},\ldots ,{\text{q}}_{541}^{T}\right)}^{T}$, ***W*** = diag(*w*_1_, *w*_2_, …, *w*_541_) and ***H***=∂***p***/∂^2^***z***_1_.

The convergent value of z21(m) gives the relative posture ^2^***z***_1_.
